# Condition dependence in biosynthesized chemical defenses of an aposematic and mimetic *Heliconius* butterfly

**DOI:** 10.1002/ece3.9041

**Published:** 2022-06-24

**Authors:** Anniina L. K. Mattila, Chris D. Jiggins, Marjo Saastamoinen

**Affiliations:** ^1^ Research Centre for Ecological Change, Organismal and Evolutionary Biology Research Programme University of Helsinki Helsinki Finland; ^2^ HiLIFE – Helsinki Institute of Life Science University of Helsinki Helsinki Finland; ^3^ Finnish Museum of Natural History (LUOMUS) University of Helsinki Helsinki Finland; ^4^ Department of Zoology University of Cambridge Cambridge UK

**Keywords:** aposematism, chemical defenses, condition dependence, cyanogenic glucosides, *Heliconius*, mimicry, trade‐offs

## Abstract

Aposematic animals advertise their toxicity or unpalatability with bright warning coloration. However, acquiring and maintaining chemical defenses can be energetically costly, and consequent associations with other important traits could shape chemical defense evolution. Here, we have tested whether chemical defenses are involved in energetic trade‐offs with other traits, or whether the levels of chemical defenses are condition dependent, by studying associations between biosynthesized cyanogenic toxicity and a suite of key life‐history and fitness traits in a *Heliconius* butterfly under a controlled laboratory setting. *Heliconius* butterflies are well known for the diversity of their warning color patterns and widespread mimicry and can both sequester the cyanogenic glucosides of their *Passiflora* host plants and biosynthesize these toxins *de novo*. We find energetically costly life‐history traits to be either unassociated or to show a general positive association with biosynthesized cyanogenic toxicity. More toxic individuals developed faster and had higher mass as adults and a tendency for increased lifespan and fecundity. These results thus indicate that toxicity level of adult butterflies may be dependent on individual condition, influenced by genetic background or earlier conditions, with maternal effects as one strong candidate mechanism. Additionally, toxicity was higher in older individuals, consistent with previous studies indicating accumulation of toxins with age. As toxicity level at death was independent of lifespan, cyanogenic glucoside compounds may have been recycled to release resources relevant for longevity in these long‐living butterflies. Understanding the origins and maintenance of variation in defenses is necessary in building a more complete picture of factors shaping the evolution of aposematic and mimetic systems.

## Graphical abstract

Understanding the origins and maintenance of variation in defenses is necessary in building a more complete picture of factors shaping the evolution of aposematic and mimetic systems. We studied associations between biosynthesized cyanogenic toxicity and a suite of key life‐history and fitness traits in a *Heliconius* butterfly under a controlled laboratory setting. Our data adds to evidence suggesting that life‐history trade‐offs involving negative associations with chemical defense traits are not particularly common in aposematic herbivores, but that variation in chemical defense levels could be introduced by condition dependent investments into defense compound production.

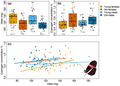

## 1 INTRODUCTION

Life‐history theory aims to explain the diversity of adaptive strategies, which organisms use in optimizing their survival and reproductive success, that is, fitness, within their environment (Flatt & Heyland, 2011; Stearns, 1992). As it is impossible to evolve optimal solutions to all selective challenges simultaneously, organisms must compromise among competing demands. Such trade‐offs, manifesting as negative correlations between different maturational, reproductive and other key traits are shaped by natural selection, which acts to maximize fitness of the life‐history as a whole. On the contrary, energetic constraints may also lead to condition dependence, where individual condition (i.e., the available resource pool of an individual; Rowe & Houle, 1996) determines the capacity to invest in multiple competing life‐history traits. For example, there is evidence from ecological studies that favorable early‐life conditions can lead to significant fitness advantages encompassing different traits in later life, often denoted as “silver spoon effects” (Grafen, 1988; Lindström, 1999; Marshall et al., 2017; Monaghan, 2008). Parental investment (passive or active condition‐transfer effects; Bonduriansky & Crean, 2018) is one important source of variation in condition. Condition dependence is expected particularly in traits that are under directional selection (fitness‐determining traits) (Andersson, 1982; Nur & Hasson, 1984). Particularly, energetically costly traits are likely candidates in trait associations related to life‐history investments. One such trait is considered to be the acquisition, production, and maintenance of chemical defenses against predation in toxic and aposematic animals (Bowers, 1992; Ruxton et al., 2018; Zvereva & Kozlov, 2016).

Aposematic animals advertise their toxicity or unpalatability to their predators, often with bright warning coloration (Cott & Hugh, 1940; Ruxton et al., 2018). The response of a predator to aposematic prey depends on the prey's relative unprofitability (Ihalainen et al., 2007; Turner, 1984) and the conspicuousness of its warning signals (Briolat et al., 2019; Gittleman & Harvey, 1980; Mappes et al., 2005). Further complexity is often introduced by the occurrence of mimicry between co‐occurring aposematic prey (Turner, 1987), which may or may not have equal defenses (Arias et al., 2016; Bowers, 1992; Ritland & Brower, 1991). From the viewpoint of the aposematic prey species, the predation pressure and the qualities of the local predator community, such as learning behavior and habitat use, are key selective agents acting on defense levels and their signaling (Briolat et al., 2019; Nokelainen et al., 2016). Evolution toward escalating defense levels would be expected if acquiring and maintaining chemical defenses were cost‐free (Cogni et al., 2012). In contrast, levels of chemical defenses often vary widely within and among species of aposematic herbivores (Arias et al., 2016; Bowers, 1992; Prudic et al., 2019; Ruxton et al., 2018; Speed, 1999; Speed et al., 2012). Explaining the mechanisms maintaining this variation is crucial in understanding the evolutionary patterns in aposematic and mimetic taxa.

Acquiring and maintaining chemical defenses could be expensive and compete for energy and raw materials (e.g., amino acids and nitrogenous compounds) with other key functions such as growth or reproduction. Consequently, in the absence of threat from predators, acquiring defenses may reduce fitness in prey species (Ruxton et al., 2018). For example, in chemically defended plants, defense costs are very common and often lead to growth‐defense trade‐offs (Cipollini et al., 2017). The allocation of energy and resources between chemical defenses and other traits could be especially tricky for aposematic herbivores, which are often caught in the middle of co‐evolutionary relationships with both the host plants on which they feed and the predator targets of their aposematic defenses (Fordyce & Nice, 2008; Lindstedt et al., 2010; Nishida, 2002; Ode, 2006). So far, the evidence for the costs of chemical defenses, related life‐history trait associations, and their role in introducing variability in chemical defenses in aposematic systems is inconclusive (Bowers, 1992; Speed et al., 2012; Zvereva & Kozlov, 2016). Defensive chemicals can either be sequestered from diet and/or biosynthesized *de novo* by the organism (Ruxton et al., 2018). A meta‐analysis by Zvereva and Kozlov (2016) examined evidence for costs of chemical defenses in 22 species of insect herbivores. The meta‐analysis indicated that *de novo* synthesis may generally be a costlier mechanism of acquiring defenses than the sequestration of plant defense compounds, which actually often seems to have even positive consequences for the performance of the herbivore (e.g., Cogni et al., 2012; Del Campo et al., 2005; Rowell‐Rahier & Pasteels, 1986; Travers‐Martin & Müller, 2007). The apparent lack of physiological costs in defense compound sequestration may be due to positive correlations between defensive and nutrient compounds in plants, as well as the high levels of adaptation in specialist herbivores to metabolize and use plant compounds as nutrients (Ruxton et al., 2018). In species able to both sequester and biosynthesize defenses, sequestration indeed seems to have energetic advantages compared with *de novo* synthesis (Zagrobelny et al., 2007). However, empirical data on species with *de novo* synthesized defenses are still very limited (Burdfield‐Steel et al., 2019; Zvereva & Kozlov, 2016), and the role of defense‐related trade‐offs in the evolution of aposematic species remains unclear (Ruxton et al., 2018).

Here, we have utilized the well‐studied system of *Heliconius* butterflies, known for the diversity of their warning color patterns and widespread mimicry (Jiggins, 2017; Merrill et al., 2015), to test the hypothesis of the costliness of *de novo* biosynthesized chemical defenses. In particular, we tested whether such costs would lead to negative associations between traits (trade‐off hypothesis), or positive associations, where the capacity to invest in defenses is dependent on individual condition (condition dependence hypothesis). *Heliconius* larvae feed on cyanogenic passion vines (*Passiflora*) (Benson et al., 1975) and can sequester the cyanogenic glucosides of some of these host plants, as well as biosynthesize the cyanogenic compounds linamarin and lotaustralin *de novo* as a chemical defense (de Castro et al., 2018, 2019; Engler et al., 2000; Engler‐Chaouat & Gilbert, 2007; Nahrstedt & Davis, 1985). The levels and chemical composition of cyanogen defenses vary widely within and among *Heliconius* species and populations (Arias et al., 2016; Cardoso & Gilbert, 2013; de Castro et al., 2019; Engler‐Chaouat & Gilbert, 2007; Mattila et al., 2021; Sculfort et al., 2020). Some of this variation is explained by a balance between plant‐derived cyanogen compound sequestration and *de novo* biosynthesis. These are negatively correlated traits in *Heliconius*, such that, on average, increased sequestration is associated with decreased *de novo* biosynthesis at species and population level, as well as at the level of individuals (de Castro et al., 2019, 2021; Engler‐Chaouat & Gilbert, 2007; Sculfort et al., 2020). Several studies have also reported evidence of unprotected automimics (Arias et al., 2016; Mattila et al., 2021; Sculfort et al., 2020), which could indicate an important evolutionary role of selection balancing costs and benefits of investing in chemical defenses (Speed et al., 2006, 2012; Svennungsen & Holen, 2007). Such wide variability in defenses could have important consequences for mimicry relationships and the evolution of aposematic coloration (Mallet & Joron, 1999; Speed, 1999). In this framework, defense variation could be a factor driving diversification in the Heliconiinae (Sculfort et al., 2020), especially since there is indication that *de novo* synthesis of cyanogenic defenses in *Heliconius* may have considerable evolutionary potential due to genetic and maternal inheritance (Mattila et al., 2021). The *Heliconius* butterflies thus provide an interesting framework to investigate defense‐related life‐history associations and their implications for aposematic and mimetic systems. To test the hypotheses of (1) the costs of cyanogen toxin biosynthesis leading to life‐history trade‐offs with other traits or (2) condition dependence in determining defense levels, we used controlled experiments in a common‐garden setting, testing associations between biosynthesized cyanogenic toxicity and a suite of important life‐history and fitness traits including growth and development, fecundity, immunity, and longevity in *Heliconius erato*.

## 2 METHODS

### 2.1 Common‐garden populations of *Heliconius erato* and host plant *Passiflora biflora*

*Heliconius erato* (Lepidoptera: Nymphalidae) is a widespread neotropical butterfly, which typically occurs together with comimetic species (usually *H. melpomene*) sharing region‐specific aposematic coloration (Supple et al., 2013). Cyanogenic toxicity of this *Passiflora* generalist (can feed on several *Passiflora*, mainly Decaloba, species such as *P. biflora*, *P. coriaceae*, and *P. auriculata* depending on the local availability of hosts; de Castro et al., 2019; Kerpel & Moreira, 2005; Merrill et al., 2013) is moderate compared with other *Heliconius* species (Arias et al., 2016; de Castro et al., 2019). It can both biosynthesize and sequester toxins, depending on the host species on which it feeds (de Castro et al., 2019; Engler et al., 2000; Engler‐Chaouat & Gilbert, 2007). Here, the larvae were reared on *Passiflora biflora*, which has complex cyanogenic compounds that *Heliconius* cannot sequester (de Castro et al., 2019). Consequently, the cyanogenic defenses of the experimental *H. erato* individuals will originate solely from *de novo* biosynthesis. *Passiflora biflora* is also the preferred host plant of *H. erato* in Panama (Merrill et al., 2013). We used a laboratory population of *H. erato demophoon* butterflies, originally established from individuals collected from wild populations in Panama (Mattila et al., 2021). The laboratory population was maintained in a greenhouse with 75% relative humidity; 8–20 h: 25°C, light; 20–8 h: 20°C, dark. The *P. biflora* used as larval host plants were likewise cultivated in common‐garden conditions to ensure uniform quality of the diet. The plants were a laboratory stock originating from *P. biflora* collected from wild populations near Gamboa, Panama. Cuttings were potted in a soil mixture (50% compost, 20% coir, 15% perlite, and 15% sand or gravel), cultivated in the greenhouse (conditions as above), and watered three times weekly (with general plant fertilizer added once weekly). All adult butterflies were marked with an individual identification number on the underside of the forewing. Butterflies were allowed to fly, mate, and oviposit freely in 2 × 2 × 2 m mesh cages with potted *P. biflora* and were provided with a standardized sugar solution diet *ad libitum* (20% sugar solution enriched with protein concentrate supplemented with amino acids; Vetark Professional Critical Care Formula; two scoops per 1 L of sugar solution) served from artificial flowers, replaced three times weekly. Eggs or early‐instar larvae were transferred into 60 × 35 × 35 cm mesh cages with cuttings of new growth of *P. biflora*, the ends of which were placed in a water container, and which were replenished three times weekly until all individuals in the cage had completed their larval development and pupated. This feeding method was chosen to reduce variation in dietary quality for experimental individuals during larval development. The larval cages were checked three times weekly for newly emerged adults, which were individually marked, their sex and eclosion time were recorded, and they were released into the main flying cage.

### 2.2 Measurements of life‐history and fitness traits

#### 2.2.1 Development

Eggs laid at a known time point were collected by placing bouquets of fresh cuttings of new growth of *P. biflora* in the flying cage of the *H. erato* common‐garden stock to allow female oviposition on the plant cuttings for a duration of 2 h. Each newly laid egg was then transferred to an individual plastic container (12 × 8 × 8 cm, closed with a mesh lid) still attached to the plant cutting (to avoid unnecessary handling). The containers were kept in the common‐garden conditions described above. Plant cuttings inside the containers were kept fresh by placing the cut ends through a hole in the lid of a small plastic cup filled with water and replacing the cuttings and water three times weekly. The containers were checked 6 days per week, once per day at around noon, to record egg hatching, pupation, and eclosion times. The newly eclosed imagines were sexed, weighed, and preserved in 100% MeOH for toxicity analysis (body excluding wings and ½ thorax; the sampled body part is not expected to influence estimates of cyanogenic glucoside compound concentrations, as these compounds are located in the hemolymph, and are thus expected to be evenly distributed throughout the body; de Castro et al., 2020).

#### 2.2.2 Body mass and wing morphology

Body mass was measured (VWR® LPC‐123, accuracy 0.001 g) from live common‐garden adult butterfly individuals belonging to two different age groups: ~1 week (median age = 7 days, *n*_female_ = 31, *n*_male_ = 30) and ~6 weeks (median age = 40 days), *n*_female_ = 28, *n*_male_ = 34). Wing morphological traits were measured from an independent dataset of young individuals, which were kept without food for 20 h before body mass and wing measurements (aged 7–10 days, *n*_females_ = 26, *n*_males_ = 26). Wings were detached from the thorax and stored in glassine envelopes before photographing them dorsally and ventrally with a DSLR camera with a 100 mm macro lens in standardized conditions. Damaged or incomplete specimens were excluded from analyses after manual inspection of images. Wing measurements were extracted from the images using custom scripts developed by Montejo‐Kovacevich et al. (2019) in image analysis software Fiji (Schindelin et al., 2012). The wing measurements included forewing perimeter (mm), total forewing area (mm^2^), wing aspect ratio (a proxy of wing shape often associated with the gliding efficiency of flight (Roy et al., 2019), corresponding here to the length of the major axis divided by the length of the minor axis, calculated by fitting an ellipse to the forewings and measuring the length of the longest axis and the length of the axis at 90° to the former), and wing loading (generally positively correlated with acceleration capacity but negatively correlated with sustained flight (Berwaerts et al., 2002), corresponding here to the ratio of adult mass per total forewing area; mg/mm^2^). Due to wing wear, the wing samples were not used for color analysis. The body excluding wings and ½ thorax were preserved in 100% MeOH for toxicity analysis.

#### 2.2.3 Immune defense

The strength of the immune defense was investigated by measuring encapsulation rate, which is a measure of a general cellular immune response which function is to form a physical barrier against, or encapsulate, a foreign object (usually a parasite or endoparasitoid) entering the body through the cuticle (Gillespie et al., 1997). Measurements were taken from common‐garden females and males of two age groups; the young age group at mean age of 8 days (*n*_FEMALES_ = 22, *n*_MALES_ = 21) and the old age group at mean age of 38 days (*n*_FEMALES_ = 18, *n*_MALES_ = 23). Butterflies were weighed preceding the encapsulation measurement. A sterile nylon monofilament of 2 ± 0.1 mm long was inserted through a puncture in the center of the live butterflies' thorax cuticle for exactly 60 min to allow an encapsulation response, after which the filament was removed and preserved in 99% EtOH. For the length of the experiment, the butterfly was secured dorsally wings opened on plastic foam with bendy plastic mesh and kept in the common‐garden conditions described above (see also Saastamoinen & Rantala, 2013). The filaments were photographed from three different angles with a microscopic camera (Nikon stereomicroscope SMZ800 with attached DS‐Fi1 camera) at standardized light conditions, and the average gray value (reflecting the strength of the encapsulation response) of the three images taken of each filament was measured and calculated using image analysis software Fiji (Schindelin et al., 2012).

#### 2.2.4 Fecundity

Following observed naturally occurring mating events in the main flying cage, newly mated females (*n* = 40, Age_mean_ = 9 days, Age_SD_ = 5 days) were transferred to separate 2 × 2 × 2 m mesh cages with potted *P. biflora*. Each female was allowed to fly, feed (standardized sugar solution diet as described above), and oviposit alone in the cage for two weeks, or until death if occurring sooner (Duration_mean_ = 11 days, Duration_SD_ = 6 days). Eggs and early‐instar larvae were collected and counted from each cage 1–2 times/ week. Consequent hatching of the eggs was not followed. In addition to the total egg number, egg laying rate was calculated as the total number of eggs divided by the total number of laying days (eggs/day). The females were weighed and preserved in 100% MeOH for toxicity analysis (body excluding wings and ½ thorax) directly following the end of each female's laying experiment.

#### 2.2.5 Age and lifespan

To test how toxicity changes with age, adult butterfly individuals were sampled at two different ages: individuals in the “young” age group were sampled at about one week after eclosion (Age_mean_ = 7.8, Age_SD_ = 2.3 days), while individuals in the “old” age group were sampled at about 6 weeks after eclosion (Age_mean_ = 38.2, Age_SD_ = 6.7 days). Samples were weighed and preserved in 100% MeOH for toxicity analysis (body excluding wings and ½ thorax). To sample the toxicity level of individuals with different lifespans, 36 common‐garden individuals (body excluding wings and ½ thorax) were preserved in 100% MeOH within the next 24 h following their natural death.

### 2.3 Butterfly toxicity analyses with ^1^H‐NMR

The concentrations of the two cyanogenic compounds biosynthesized by *Heliconius* larvae and adults, linamarin and lotaustralin, were analyzed from the butterfly samples using nuclear magnetic resonance (^1^H‐NMR). Sequestered compounds were not recorded, as their presence was not expected due to the incapability of *H. erato* to sequester cyanogenic compounds of *P. biflora* (de Castro et al., 2019; see above). Samples may have included cyanogenic compounds of more complicated structure originating from ingested *P. biflora* (de Castro et al., 2019); however, the method allowed reliable identification and quantification of the exact biosynthesized compounds of interest. Sample extraction and ^1^H‐NMR analysis followed the procedures of Mattila et al. (2021), applying methodology described in Kim et al. (2010). Samples were dried, weighed, homogenized (TissueLyser, Qiagen; 30/s, 4 × 20 s) in an extraction/NMR solvent consisting of 400 μl KH_2_PO_4_ buffer in D_2_O (pH 6.0) containing 0.1% (wt/wt) 3‐(trimethylsilyl)propionic‐2,2,3,3‐d_4_ acid sodium salt (TSP) and 400 μl Methanol–d_4_ (CD_3_OD) 99.8%, vortexed for 1 min, sonicated for 20 min (Eurosonic 22), and centrifuged 15 min at 13.2 × 10^3^ rpm, before pipetting 625 μl of supernatant into a high‐throughput NMR tube (Wilmad WG‐1000‐7 5*177.8 mm). ^1^H‐NMR was performed at the Finnish Biological NMR Center using Bruker Avance III HD NMR spectrometer (Bruker BioSpin, Germany) equipped with a cryogenic probe head and operated at ^1^H frequency of 850.4 MHz at 25°C. A standard cpmgpr1d pulse program (Bruker BioSpin; Carr & Purcell, 1954) was used, with 32 scans, 32,768 data points, a spectral width of 10,204 Hz and a relaxation delay of 3 s. The data were processed and analyzed using Bruker TopSpin software (versions 3.2pl6 and 3.5pl6) as in Mattila et al. (2021). The peaks of CNglcs compounds were identified based on comparison of sample ^1^H spectra with the ^1^H spectra of authentic reference samples of linamarin and lotaustralin. Concentrations of the CNglcs compounds were calculated based on the extracted areas of singlet peaks at 1.652 ppm (epilotaustralin) and 1.668 ppm (lotaustralin), and a duplet peak at 1.698 ppm (linamarin), quantified based on the extracted area of the calibration compound 0.1% (wt/wt) 3‐(trimethylsilyl)propionic‐2,2,3,3‐d4 acid sodium salt (TSP), and corrected for the sample dry mass.

### 2.4 Statistical analyses

Variables were checked for normality and collinearity. Developmental time (larval time from egg hatching to pupation and total development time from egg to adult) and lifespan data were LOG‐transformed preceding further analyses. General linear models and ANOVA were applied to test for associations between the concentration of cyanogenic toxins (the combined concentration of linamarin and lotaustralin) and the studied life‐history and fitness traits in R 3.6.1 (R Core Team, 2018). If not otherwise noted, models included the trait, sex, and their interaction as explanatory variables.

## 3 RESULTS

### 3.1 Cyanogen concentration

The mean concentration of biosynthesized cyanogenic glucoside compounds in all samples was 0.936% and 0.908% in female and male adult butterflies, respectively (Table 1). The toxicity levels did not differ between the two sexes (*t*_292.36_ = 0.686, *p* = .493).

**TABLE 1:** Summary statistics of the total concentration of biosynthesized cyanogen compounds (linamarin + lotaustralin; % dry mass) in female and male *H. erato* adult butterflies

Sex	Min.	Median	Mean	Max.	*n*
Female	0.194	0.929	0.936	2.064	182
Male	0.262	0.840	0.908	1.908	136

### 3.2 Development

**FIGURE 1:**
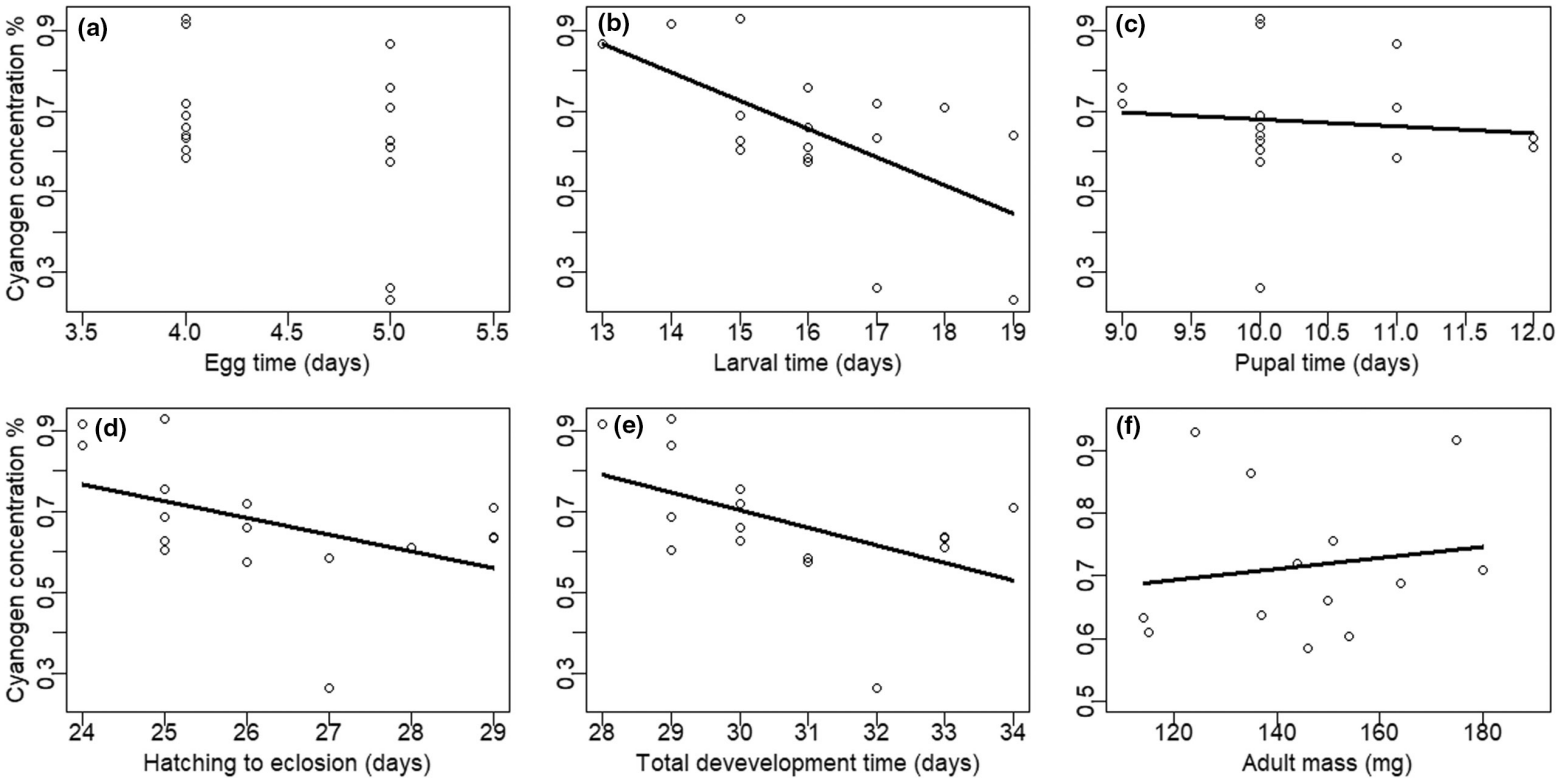
Association of biosynthesized cyanogen toxin concentration (% dry mass) with development times. (a) Number of days from egg laying to egg hatching. (b) Number of days from egg hatching to pupation. (c) Number of days from pupation to emergence of adult butterfly. (d) Number of days from egg hatching to emergence of adult butterfly. (e) Number of days from egg laying to emergence of adult butterfly. (f) Mass of adult butterfly (mg)

In terms of total development rates (log‐transformed time from egg to adult), our data indicate that individuals with higher concentrations of cyanogenic toxins develop at a faster pace (*F*_1,14_ = 4.775, *p* = .046; Figure 1), without compromising on their final adult mass (final adult mass was independent of cyanogen toxin concentration; *F*_1,11_ = 0.267, *p* = .616; Figure 1). When breaking the development time into the different stages, it was evident that egg and pupal development times were not associated with toxicity levels (*F*_1,15_ = 2.154, *p* = .163; *F*_1,14_ = 0.134, *p* = .720 for egg and pupal times, respectively; Figure 1), while larval development was faster in more toxic individuals (*F*_1,15_ = 8.769, *p* = .010; Figure 1).

### 3.3 Body mass and wing morphology

**FIGURE 2:**
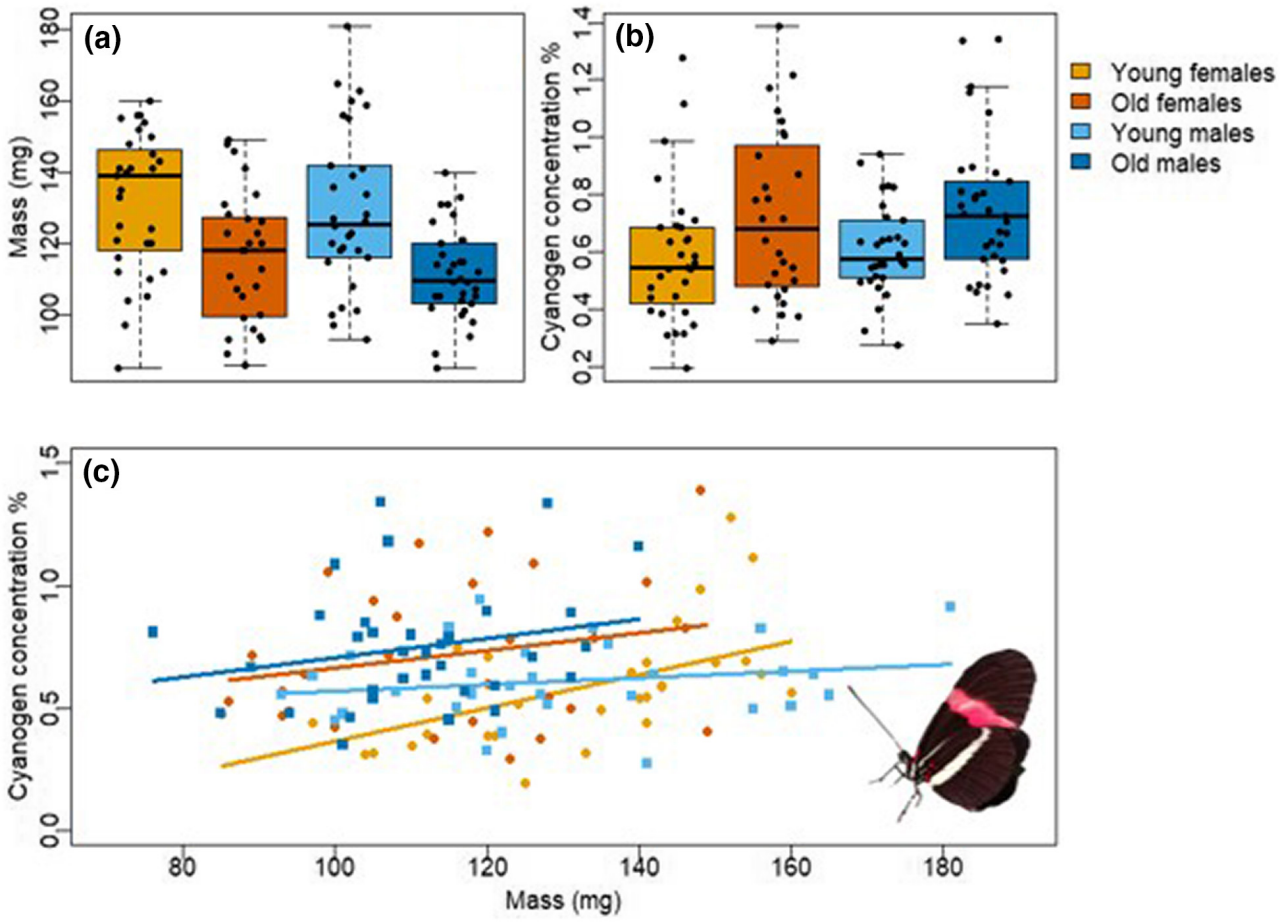
Body mass and biosynthesized cyanogen toxicity in young (1 week) and old (6 weeks) age groups of *Heliconius erato*. (a) Body mass (mg), (b) biosynthesized cyanogen concentration (% of dry mass), and (c) association of cyanogen concentration and body mass in young and old females and males

Females and males did not differ in body mass in either age group (*F*_1,119_ = 1.21, *p* = .27), but young individuals were heavier than old individuals in both sexes (*F*_1,119_ = 27.3, *p* < .001; Figure 2a). In female and male butterflies, old individuals were significantly more toxic than young individuals (*F*_1,119_ = 10.9, *p* = .001; Figure 2b). The total cyanogen concentration was not associated with body mass in the old age group (*F*_1,58_ = 2.68, *p* = .107; Figure 2c). In contrast, in the young age group, cyanogen concentration was positively associated with body mass (*F*_1,57_ = 11.3, *p* = .001; Figure 2c), especially in females (body mass:sex‐interaction *F*_1,57_ = 5.96, *p* = .018; Figure 2c). None of the wing morphology measures, including wing area, wing loading, and wing aspect ratio, were associated with cyanogenic toxicity (Appendix S1).

### 3.4 Immune defense

**FIGURE 3:**
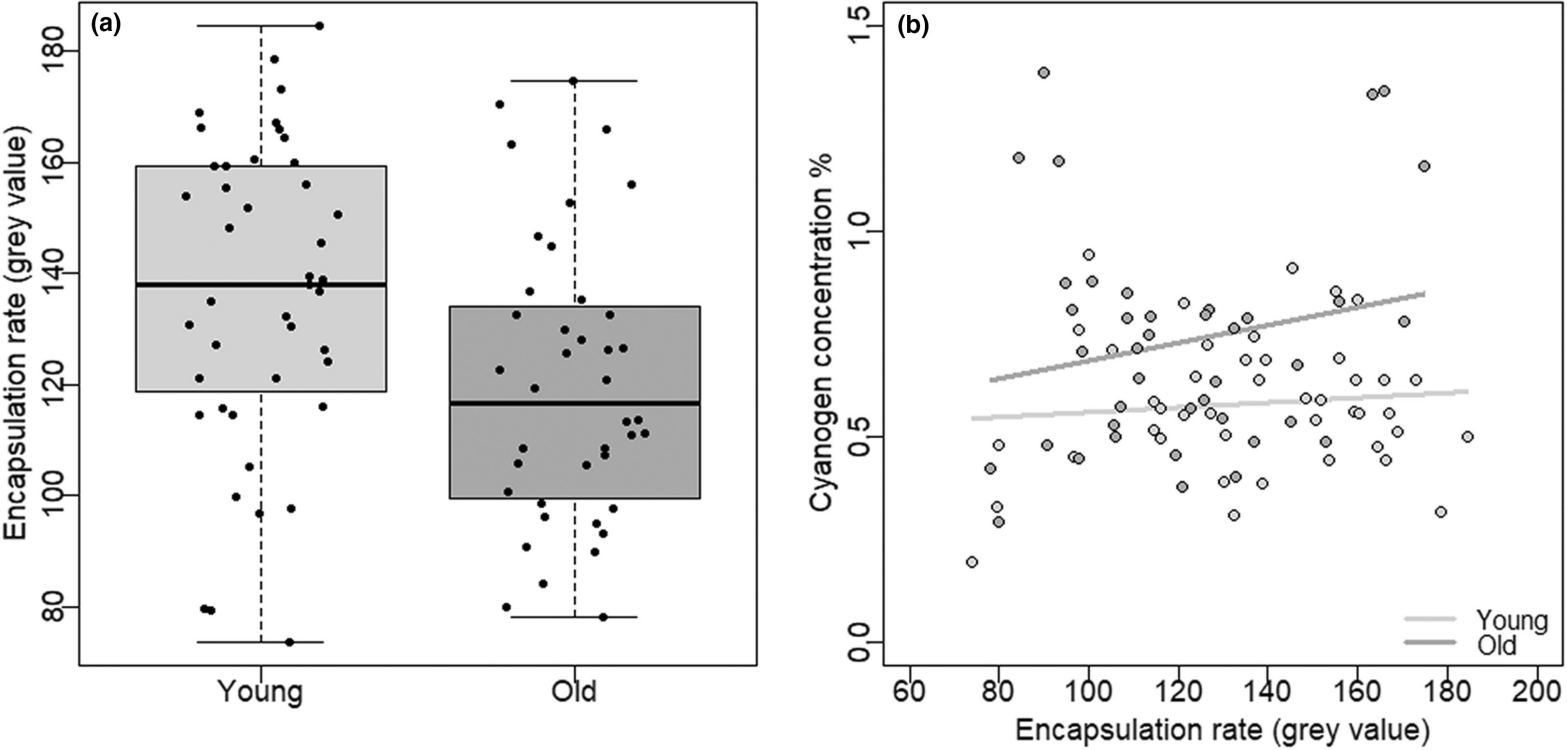
(a) Encapsulation rate (average gray value of encapsulation tissue formed in one hour around a foreign object inserted into the body) in young (1 week) and old (6 weeks) individuals. (b) Association of encapsulation rate with biosynthesized cyanogen concentration (% dry mass) in old and young individuals

We used the rate of encapsulation of a foreign object at adult stage as a measure of general immune defense status. Encapsulation rate was significantly higher in the young age group irrespective of sex (*F*_1,79_ = 7.724, *p* = .007; Figure 3a), but it was not associated with cyanogen concentration in either sex or age group (*F*_1,78_ = 0.178, *p* = .67; Figure 3b).

### 3.5 Fecundity

**FIGURE 4:**
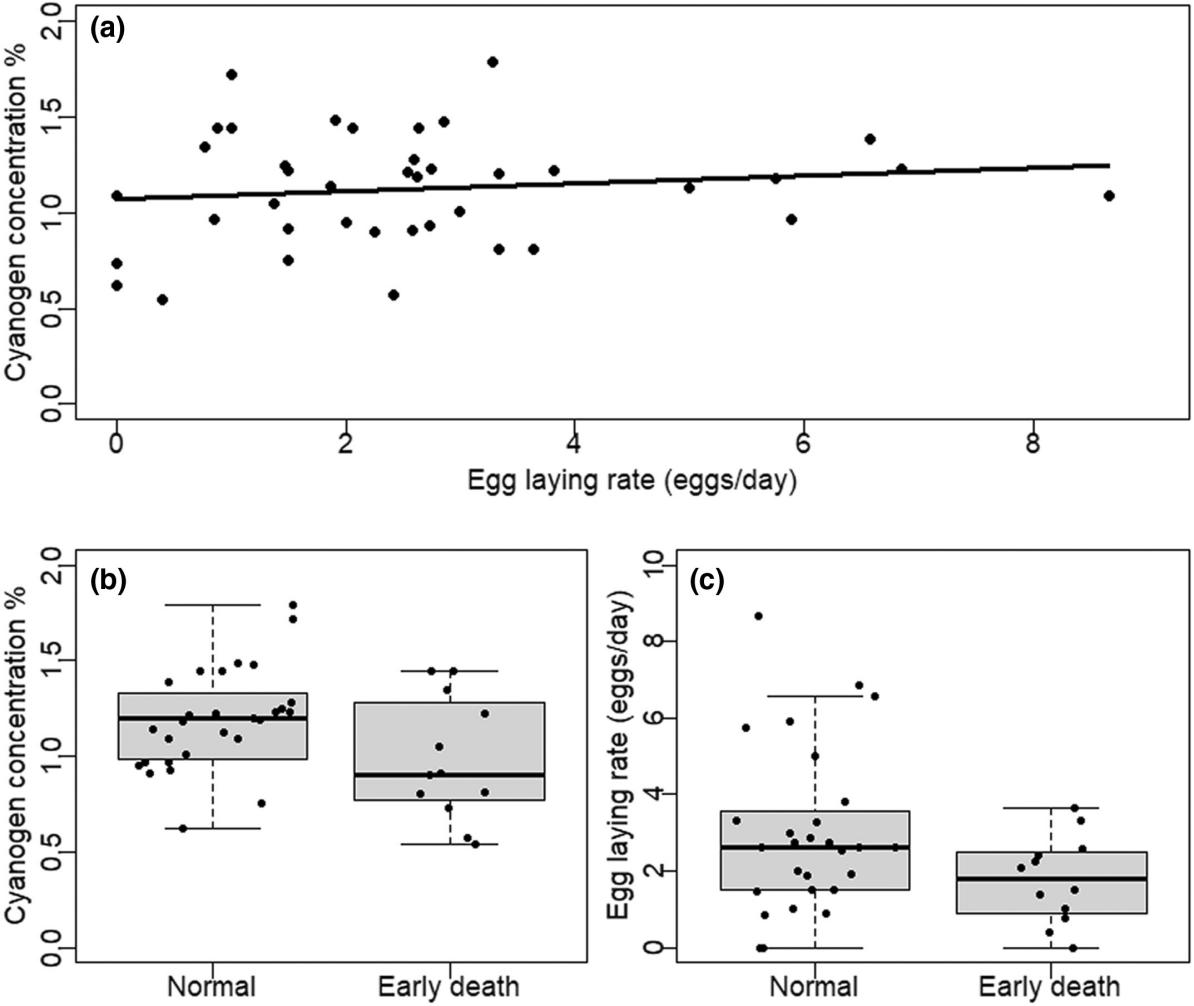
(a) Association of biosynthesized cyanogen concentration (% of dry mass) with female egg laying rate (eggs/day). (b) Biosynthesized cyanogen concentration (% of dry mass) and (c) egg laying rate (eggs/day) in females that continued to lay eggs until the end of the experiment (“Normal”), and in those which died at a young age during the experiment (“Early death”)

We used egg laying rate (number of eggs laid/day) as a proxy of female fecundity, because the duration of time the egg laying was observed varied among females (Mean = 11, *SD* = 6 days). Female body mass was not associated with egg laying rate (*F*_1,17_ = 0.565, *p* = .462) or cyanogen toxicity (*F*_1,17_ = 0.766, *p* = .394) and was not included in further models. The age of females at the start of the egg laying experiment was similar (Mean = 8.8, *SD* = 4.8 days) and was not associated with egg laying rate. Further, female age at sampling was similar (Mean = 19.9, *SD* = 7.8 days) and was not associated with their cyanogen toxin concentration (*F*_1,38_ = 1.145, *p* = .291). There was no significant association of cyanogen toxicity level with female egg laying rate (*F*_1,38_ = 0.678, *p* = .416; Figure 4a). However, 30% of the females died during the period in which their egg laying rate was observed. These females had on average lower cyanogen toxicity level (*F*_1,38_ = 4.593, *p* = .039: Figure 4b) and tended to have lower egg laying rate (not statistically significant; *F*_1,38_ = 3.465, *p* = .070; Figure 4c) than those females that continued to lay eggs until the end of the experiment.

### 3.6 Age and longevity

**FIGURE 5:**
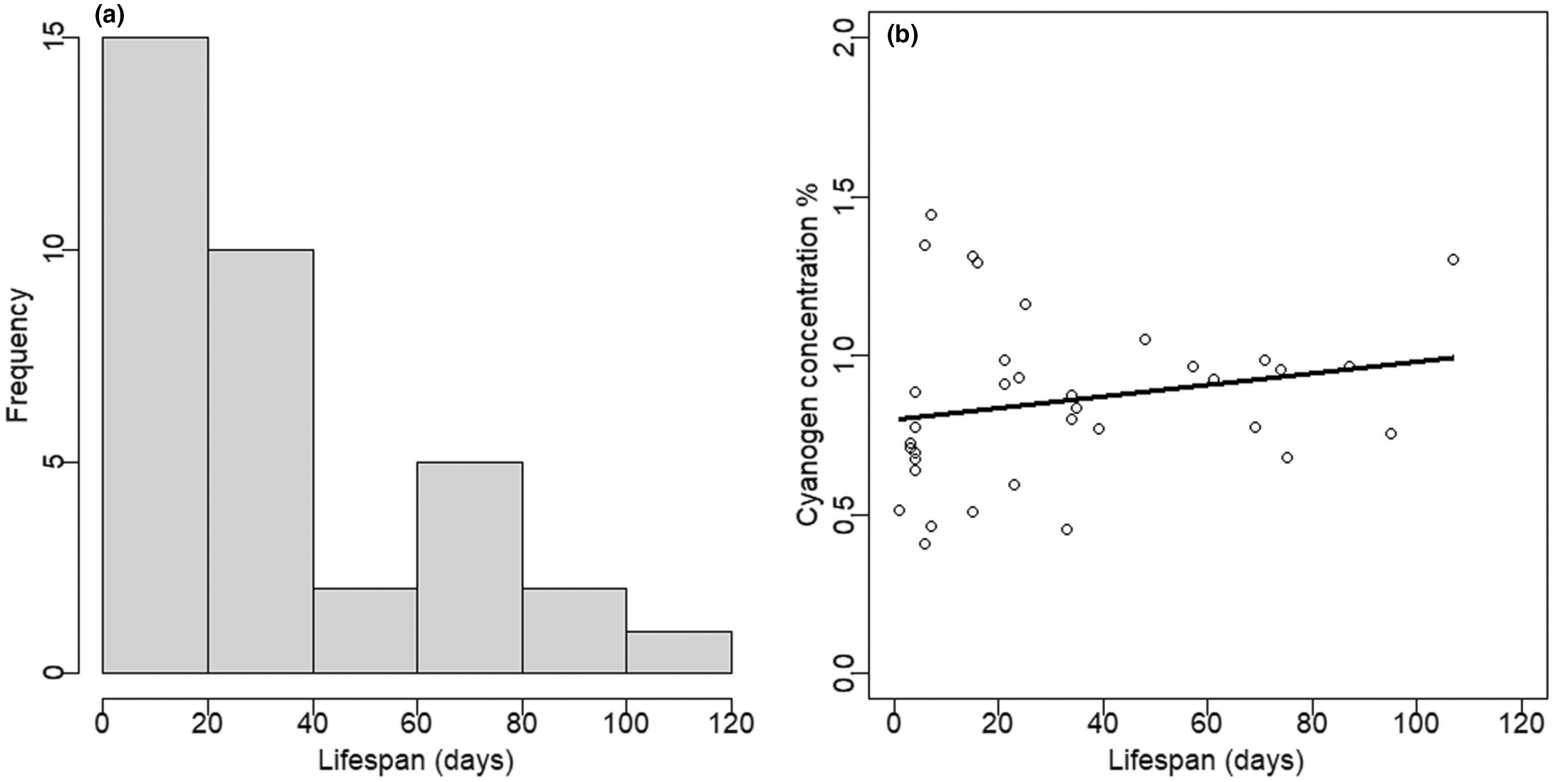
(a) Distribution of observed lifespans. (b) The association of biosynthesized cyanogen concentration (% dry mass, measured at the time of natural death) with lifespan

Unlike in individuals tested for cyanogen toxicity level at either young (1 week) or old (6 weeks) age, in which the old individuals had significantly higher cyanogen concentrations than young individuals (*F*_1,119_ = 10.9, *p* = .001; Figure 1b), in the individuals tested for toxicity after their natural deaths, cyanogen concentration was independent of lifespan (*F*_1,31_ = 2.456, *p* = .127 for log‐transformed data; Figure 5b). Here, cyanogen concentration was similar in individuals naturally dying after substantially different lifespans, which ranged here from 1 to 107 days (distribution of lifespans, see Figure 5a). Lifespan (or its association with cyanogen toxicity) did not differ between the two sexes (*F*_1,31_ = 0.181, *p* = .673), with females and males having average lifespans of 30 and 37 days, respectively. On the contrary, there is some indication that the likelihood of dying at a young age may be greater in individuals with low cyanogen toxicity, at least in mated females, in which individuals with on average low cyanogen toxicity level more often stopped laying eggs and died at a young age (*F*_1,38_ = 4.59, *p* = .039; Figure 2b).

## 4 DISCUSSION

### 4.1 Life‐history trade‐offs involving chemical defenses are not common in aposematic species

The complex selection schemes acting on chemically defended aposematic animals (Briolat et al., 2019) make them an interesting case for studying life‐history evolution. The allocation of energy and resources could be especially constrained in these species, leading to trade‐offs between the possibly costly production and maintenance of defensive chemicals and other expensive life‐history traits (Ruxton et al., 2018; Zvereva & Kozlov, 2016). Here, we investigated a suite of energetically expensive key life‐history traits and their associations with biosynthesized cyanogenic toxicity in an aposematic *Heliconius* butterfly and found no indication of trade‐offs. In contrast, we found life‐history traits to be either unassociated or positively associated with the level of chemical defenses.

Our result is contradictory with some studies on aposematic insects that find costs associated with chemical defenses. For instance, in the aposematic wood tiger moth *Arctia plantaginis*, the excretion of its defensive fluid (which contains also *de novo* synthesized compounds; Burdfield‐Steel et al., 2018) has negative consequences for reproductive output (Lindstedt et al., 2020). Handling/detoxifying of defensive compounds in its plant diet also comes at a cost to developmental traits and warning signal expression (Lindstedt et al., 2010; Reudler et al., 2015). Similarly, the fecundity of cardenolide‐sequestering *Oncopeltus fasciatus* milkweed bugs was reduced when constantly artificially maintained on cardenolide‐containing diet, although in general, cardenolide exposure was associated with positive fitness effects (Pokharel et al., 2021).In the gregarious social *Diprion pini* pine sawfly, increased allocation to defensive secretion incurs costs in growth, immunity, and survival (Björkman & Larsson, 1991; Lindstedt et al., 2018), and in the pipevine swallowtail (*Battus philenor*) butterfly, toxin content is negatively correlated with fat content (Fordyce & Nice, 2008). Most evidence of defense costs is from the very few studies on species that biosynthesize their own chemical defenses, in contrast to the more numerous studies on species which sequester the defensive compounds of their host plants and often show neutral or positive fitness consequences of increasing defense levels (reviewed by: Bowers, 1992; Ruxton et al., 2018; Zvereva & Kozlov, 2016). In particular, these data not only suggest that physiological costs of *de novo* synthesis of defensive compounds may generally be higher than the costs of sequestered defenses, but also that this could be true especially in the case of external secretion of defensive substances (Zvereva & Kozlov, 2016). The more recent results on, for example, the *A. plantaginis* study system seem to support this (Burdfield‐Steel et al., 2019; Lindstedt et al., 2020). In this framework, our results add to evidence that sequestration and *de novo* synthesis of defensive compounds, with the potential exception of externally secreted substances, may generally not lead to trade‐offs with key life‐history and fitness traits in aposematic herbivores. Our results are also in line with observations from meta‐analyses indicating general beneficial effects of plant secondary metabolites on herbivore performance (Smilanich et al., 2016) and that positive interactions (instead of trade‐offs) in host‐use adaptations form a dominant mechanism for host‐use evolution in herbivorous insects (Peterson et al., 2016).

Our results indicated that many energy‐expensive key traits are positively correlated with biosynthesized cyanogen toxicity levels. More toxic individuals had faster development and higher body mass and a tendency for increased survival and fecundity. Together, these effects could result in significant increases in cyanogen level with increasing fitness (and vice versa). One explanation for this result is that cyanogenic toxicity or the capacity for cyanogen biosynthesis is correlated with overall physiological condition, dependent on, for example, the resource pool, genetic background, and earlier conditions experienced by the individual, in line with our condition dependence hypothesis. In our study, individuals developed in common‐garden conditions (and thus environmental sources of variation in physiological condition are expected to be minimal), but differential maternal investments could have caused individual differences in early‐life conditions. In particular, maternal investments in offspring chemical defense level could have played a role; *Heliconius* females are known to deposit cyanogenic glucoside toxins into eggs (Nahrstedt & Davis, 1983), and a previous study indicated a central role for maternal effects in the inheritance of biosynthesized cyanogenic toxicity (Mattila et al., 2021). It is unclear whether a higher cyanogen concentration in eggs is directly associated with fitness benefits, a question that could be addressed by future studies investigating individuals of known maternal identity (and thus, average maternal egg toxin level). However, there is indication that maternally contributed cyanogenic compounds are utilized in the growth and development of early‐instar *Heliconius* larvae (de Castro et al., 2019), which could explain “silver spoons effects” encompassing biosynthesized toxicity as well as other life‐history traits in later life. Such condition dependence in *de novo* production of defensive compounds (investing in defenses only given sufficient resources) may be common in aposematic herbivores. One known example comes from ladybird beetles: increased dietary resources increase *de novo* production of defensive compounds in *Coccinella septempunctata* (Blount et al., 2012), and the alkaloid recovery rate after reflex bleeding is faster in *Adalia bipunctata* individuals feeding on a high‐quality diet (Oudendijk & Sloggett, 2021). In the wood tiger moth *Arctia plantaginis*, the toxicity level of its defensive substance is dependent on the availability of dietary resources (Burdfield‐Steel et al., 2019).

It should be noted that empirical studies of trade‐offs using investigations of pure phenotypic correlations in unmanipulated individuals in the field or laboratory can provide important clues about actual physiological trait associations and their causes, but they have important shortcomings (Zera & Harshman, 2001). For example, uncontrolled variables can reduce the magnitude or direction of correlations between traits that are actually involved in a trade‐off. Perhaps most importantly, individual variation in nutrient intake as well as the absolute amount of nutrient input can substantially influence the phenotypic expression of trade‐offs; for example, increased input can diminish or “hide” an apparent trade‐off (Zera et al., 1998; Zera & Harshman, 2001). Variable nutrient intake can also cause two traits that trade‐off with each other to be seemingly positively correlated (de Jong, 1993; Zera & Harshman, 2001). This is also a potential explanation for neutral or positive correlations with defensive traits. Our experiment was carried out in benign common‐garden conditions with ad libitum diet, which we expect to provide a relatively controlled setting in terms of nutrient input and environmental stressors. However, the unlimited dietary resources and lack of environmental stressors could have also masked some trait correlations which could prove negative in a situation with nutrient limitations. Future studies of defensive traits should take steps toward controlled and/or manipulated nutrient inputs, as well as explicitly investigate the physiological mechanisms of the observed phenotypic correlations. Furthermore, in species capable of both *de novo* biosynthesis and sequestration, controlled comparison of costs between these two mechanisms will also be of future interest.

Our data add to evidence suggesting that life‐history trade‐offs involving negative associations with chemical defense traits are not particularly common in aposematic herbivores (except for the potential exception of external secretion of defensive substances; Burdfield‐Steel et al., 2019; Lindstedt et al., 2020; Zvereva & Kozlov, 2016), but that variation in chemical defense levels could be introduced by condition‐dependent investments into defense compound production. Costs of producing and maintaining toxicity may result in frequency‐ or density‐dependent selection in resource optimization and warning‐signal honesty (Blount et al., 2009, 2012; María Arenas et al., 2015; Speed et al., 2012). The existence of non‐protected “cheaters” or so‐called automimics (Brower et al., 1970) has been documented in a diverse array of chemically protected taxa (Ruxton et al., 2018; Speed et al., 2012), including *Heliconius* (Arias et al., 2016; Mattila et al., 2021; Sculfort et al., 2020). These automimics gain protection via their defended conspecifics without investing in defenses themselves (Ruxton et al., 2018; Speed et al., 2012) and may dilute the protection given by the warning signal as in Batesian and quasi‐Batesian mimicry (Gibson, 1984; Jones et al., 2016; Speed et al., 2012). Such cheating is expected to be beneficial if defenses protect individuals during attack (taste‐rejection; Skelhorn & Rowe, 2006) but are, on the contrary, associated with a significant cost (Speed et al., 2006; Svennungsen & Holen, 2007). The occurrence of automimicry thus suggests that despite the apparent lack of defense‐trait‐related trade‐offs, associated costs could play a role in the evolution of aposematic species.

In such cases, in which no clear costs of a defense toxin can be demonstrated, the escalation of defense levels could still be prevented by several selection mechanisms. For example, variation in toxin levels could still be high if the efficacy of the toxin on the target predators rapidly saturates (Speed et al., 2012). There is indication that this could be true for the efficacy of *Heliconius* cyanogen defenses on some bird predators (Chouteau et al., 2019). Variation in defenses can also be maintained by predators responding not only to average defense levels, but to the variation in them (Barnett et al., 2014; Skelhorn & Rowe, 2005).

### 4.2 The role of other life‐history correlates of chemical defenses

Finally, even outside of life‐history trait correlations related to energetic costs of production and maintenance of chemical defenses, there may be life‐history‐related explanations for toxin diversity, such as effects of age and maturity, and the competition of molecules used in additional roles such as communication (Speed et al., 2012). Our data suggest some potentially important interactions of cyanogenic toxins with life‐history functions and their variation in *Heliconius*. Older adult individuals were more toxic on average compared with newly eclosed adults, supporting previous findings of accumulation of cyanogenic toxins with age in *Heliconius* (de Castro et al., 2020; Nahrstedt & Davis, 1985).

Furthermore, there is evidence that cyanogenic compounds can be catabolized to recycle nitrogen and glucose, which could be further utilized in other life‐history functions (de Castro et al., 2020). Our data indicating that cyanogenic toxicity at the time of natural death is similar in individuals with highly different lifespans support the possibility of such recycling as a “final resource” at the end of life. Long‐living individuals may have enabled their longer lifespan by recycling cyanogenic compounds, which could explain why individuals that died at substantially older age no longer expressed the pattern of accumulated toxins with age. An increase in lifespan by recycling of defensive compounds could have a substantial positive influence also on lifetime reproductive fitness in *Heliconius*, which continue to reproduce throughout their life (Dunlap‐Pianka et al., 1977). Both continuous reproduction and the extraordinarily long lifespan in comparison with most other Lepidoptera are thought to be mostly enabled by pollen feeding in *Heliconius* (Gilbert, 1972). Our study supports the suggestion that in addition to the key influence of pollen feeding, cyanogen compounds play an important role in the cycling of energy and resources in *Heliconius* (Cardoso & Gilbert, 2013; de Castro et al., 2020).

Elucidating the physiological mechanisms and sources of variation in chemical defenses and interactions with key life‐history traits is a major step in gaining a more complete understanding of toxin diversity. Such knowledge advances our understanding of the complicated dynamics of selection pressures on chemical defenses, and the implications of such dynamics for the evolution and diversification of aposematic and mimetic species (Briolat et al., 2019; Speed et al., 2012).

## AUTHOR CONTRIBUTIONS

**Anniina L. K. Mattila:** Conceptualization (equal); data curation (lead); formal analysis (lead); funding acquisition (lead); investigation (lead); methodology (lead); project administration (lead); resources (equal); visualization (lead); writing – original draft (lead); writing – review and editing (equal). **Chris Jiggins:** Conceptualization (equal); supervision (equal); writing – review and editing (equal). **Marjo Saastamoinen:** Conceptualization (equal); supervision (equal); writing – review and editing (equal).

## CONFLICT OF INTEREST

The authors declare no competing interests.

## Supporting information


Appendix S1

## Data Availability

All data are openly available in the public data repository Dryad; DOI: 10.5061/dryad.hmgqnk9kh. All wing images are available in the public repository Zenodo; DOI: 10.5281/zenodo.2555086.
