# Supplementary material for: Condition dependence in biosynthesized chemical defenses of an aposematic and mimetic Heliconius butterfly
Source: Ecol Evol. 2022 Jun 24;12(6):e9041. doi: 10.1002/ece3.9041 (PMC9227709; doi:10.1002/ece3.9041)
Supplement: Supplementary file 1 — Appendix S1 [file ECE3-12-e9041-s001.docx]

**APPENDIX**

**Appendix 1:** **Wing morpology.**

Wing morphology and its association with cyanogen toxicity was analyzed in an independent dataset of young individuals (aged 7-10 days, *n*_females_ = 26, *n*_males_ = 26). Here, females were heavier than males (*F*_48,1_ = 11.54, *P* = 0.001), and body mass was not significantly associated with cyanogen toxicity (*F*_48,1_ = 1.47, *P* = 0.23). Wing area and wing perimeter were positively correlated with each other (*Pearson* *cor* = 0.96, *P* <0.01) and with body mass (*Pearson* *cor* = 0.72, *P* <0.01 and *Pearson* *cor* = 0.76, *P* <0.01 for area and perimeter, respectively). Also wing loading was positively correlated with body mass (*Pearson* *cor* = 0.87, *P* <0.01). Wing areas were similar in females and males (*F*_48,1_ = 0.48, *P* = 0.49), and due to heavier body mass females had on average higher wing loading than males (*F*_48,1_ = 19.48, *P* < 0.001). None of the wing measures collinear with body mass were significantly associated with cyanogen concentration (Fig. Appendix 1). Aspect ratio was the only measure of wing morphology independent of body mass (*Pearson* *cor* = -0.15, *P* = 0.28). This wing shape measure did not differ between the sexes (*F*_48,1_ = 1.75, *P* = 0.19) and it was not associated with cyanogen concentration (*F*_48,1_ = 2.17, *P* = 0.15: Fig. Appendix 1).


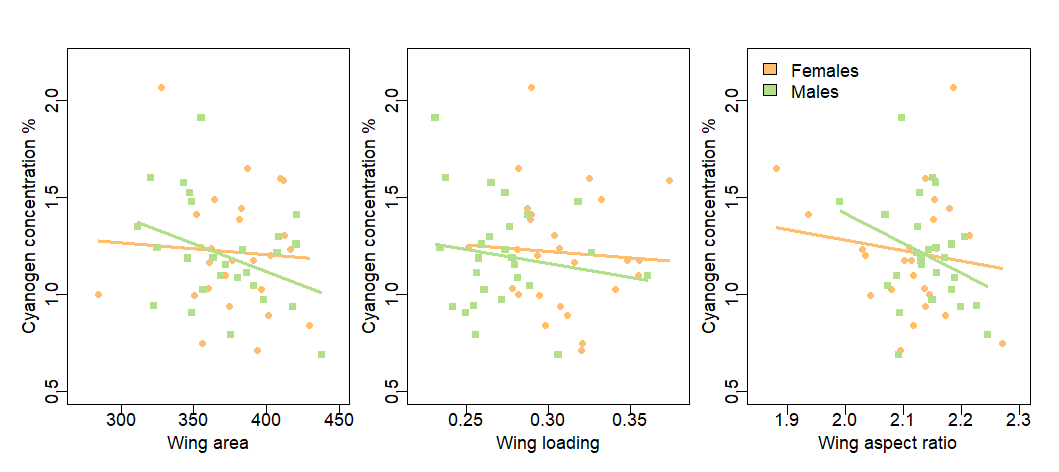


**Figure Appendix 1.** The association of key wing morphology traits forewing area (mm^2^), wing loading (mg/mm^2^), and aspect ratio with biosynthesized cyanogen concentration (% of dry mass).
